# Effects of a Smartphone-Based Positive Reflection Diary on Work Engagement Among Japanese Workers: Randomized Controlled Trial

**DOI:** 10.2196/55664

**Published:** 2025-09-25

**Authors:** Masahito Tokita, Shuichiro Kobayashi, Daisuke Miyanaka, Nobuyuki Takate, Hiroyuki Nakano, Kazuki Takeuchi, Akihito Shimazu

**Affiliations:** 1 Keio University, Keio Research Institute at SFC, Fujisawa, Japan; 2 Faculty of Environment and Information Studies, Keio University, Fujisawa, Japan; 3 Better Options, Inc, Tokyo, Japan; 4 Faculty of Policy Management, Keio University, 5322 Endo, Fujisawa, 252-2882, Japan, 81 466483165; 5 Japan Productivity Center, Tokyo, Japan; 6 Labor Trust LCC, Tokyo, Japan

**Keywords:** work engagement, positive reflection, self-monitoring, smartphone-based diary, randomized controlled trial

## Abstract

**Abstract:**

**Background:**

Work engagement is an important determinant of workers’ well-being. According to the job demands–resources model, personal resources are one of the key antecedents of work engagement. Enhancing personal resources leads to improved work engagement. Furthermore, reflecting positively on one’s achievements at work may enhance personal resources. Hence, there is a need for a simple, self-guided tool such as a smartphone app that can be used by employees to record and reflect on their work accomplishments.

**Objective:**

This study aimed to evaluate the effect of a smartphone-based diary (ie, Work Engagement Diary) that promotes positive reflection in daily working life on work engagement among Japanese workers in a randomized controlled trial.

**Methods:**

Six hundred Japanese workers who met the inclusion criteria were randomly allocated to either the intervention group or the wait-list control group (300 participants each) via a web survey company. Participants in the intervention group kept the Work Engagement Diary for 2 weeks, whereby they were required to set a weekly goal at the beginning of the week and fill in their work achievements at the end of the day. Those in the waitlist control group did not receive any interventions until they completed a follow-up survey. Work engagement was assessed at preintervention (T1), postintervention (T2), and 3 weeks after intervention (T3) in both groups.

**Results:**

A mixed model for repeated measures conditional growth model analysis using intention-to-treat revealed a significant improvement in work engagement for the intervention group compared to the wait-list control group throughout the study period (*P*=.04). Effect sizes were small in work engagement for T1 versus T2 (Cohen *d*=0.11 [95% CI −0.06 to 0.28]) and for T1 versus T3 (Cohen *d*=0.12 [95% CI −0.06 to 0.28]).

**Conclusions:**

This randomized controlled trial demonstrated that our newly developed smartphone-based positive reflection diary at work effectively improved work engagement among Japanese workers. Future research needs to clarify longer-term intervention effects and detailed mechanisms of the intervention effects.

## Introduction

### Work Engagement

Work engagement is beneficial for employees and organizations alike [1,2] and is associated with work- and health-related positive outcomes. Work engagement refers to employees’ strength and optimal functioning and positive work-related states characterized by vigor, dedication, and absorption in their work [3]. Vigor is defined as high levels of energy and mental resilience in one’s work. Dedication is a strong involvement in one’s work combined with a sense of significance, enthusiasm, and challenge. Absorption is defined as having higher concentration and being happily engrossed in one’s work. It is viewed as a psychological state in which time seems to pass quickly and it is difficult to leave work [1]. A recent meta-analysis [4] reported that work engagement was positively related to job performance, job satisfaction, and organizational commitment and negatively related to turnover intention. Furthermore, work engagement as an antecedent of health- and work-related outcomes is positively associated with variables such as job performance and job satisfaction and negatively associated with variables such as work withdrawal behavior and physical and mental ill health [5,6].

### Job Demands–Resources Model

According to the job demands–resources (JD-R) model [1,7], job resources foster work engagement in a positive motivational process wherein an increase in job resources leads to an increase in work engagement and positive health and organizational outcomes. On the contrary, job demands cause exhaustion in an energy-sapping process [8,9] wherein an increase in job demands (such as stressors) leads to an increase in burnout and negative health and organizational outcomes. Personal resources (such as positive emotions, vitality, and mental acuity) as antecedents have been included in the extension of the JD-R model and may be directly or indirectly related to work engagement [10-12]. Positive work reflection is considered a personal resource in the JD-R model and plays a key role in enhancing work engagement [4]. Research suggests that when employees reflect on their work achievements and growth, their personal resources increase, leading to higher work engagement [13]. Furthermore, positive reflection helps sustain work engagement—even under high job demand—highlighting its importance in promoting work engagement [14]. Therefore, interventions that focus on increasing personal resources, such as focusing on what workers have achieved rather than what they have not achieved, may improve work engagement.

### Positive Reflection

Increasing awareness of one’s own achievements at work leads to positive health outcomes among workers [12]. Reflecting positively on one’s work, which focuses on achievement at work, leads to a reevaluation of potentially stressful work situations [15]. Positive work reflection refers to thinking about the positive aspects of one’s job and includes thoughts about pleasurable events such as successful task performance and supportive work relationships [16]. A recent meta-analysis [17] reported that off-job positive work-related thoughts had a positive relationship with work engagement (ρ=0.493, 95% CI 0.407-0.579). Thus, positive reflection about one’s achievement at work during off-job time can lead to improved work engagement. Furthermore, self-monitoring may be helpful for reflecting positively on one’s work. Keeping a diary of positive work achievements, including increasing access to positive emotions, and positive work-related thoughts can be a strategy for self-monitoring. According to social cognitive theory, self-monitoring influences people’s motivation and behavior by increasing their interest in their actions and their occurrence and effects [18,19].

### Smartphone-Based Apps

There are two types of smartphone apps: those that run on a web browser (web apps) and those that run as native apps. In general, native apps are considered to have an advantage over web apps in terms of CPU and memory usage [20]. However, few health-related native apps are supported by evidence [21]. Paganin and Simbula [22] reviewed 31 studies that examined the impact of workplace smartphone-based apps on improving employees’ physical health, targeted symptoms (eg, depressive symptoms), and well-being. Of the eight interventions specifically designed to improve worker well-being, only four—namely, Headspace, Hello Mindcare, Viary, and Smartphone Resiliency Training—were strictly native smartphone apps. The other four were intervention programs accessible via web browsers compatible with smartphones. These findings suggest that native smartphone apps explicitly designed to improve worker well-being are limited.

In a meta-analysis of intervention studies designed to improve work engagement among employees published between 2009 and 2017 [23], 15 online (web-based or web apps) intervention studies were included, yielding an effect size of 0.14 (*P*<.05). Furthermore, while app stores offer numerous health-related apps claiming to improve well-being, only a small proportion of them have undergone rigorous scientific evaluation. According to Lau et al [24], of the 1009 psychosocial wellness and stress management smartphone apps available for self-help, only 21 (2.08%) were supported by peer-reviewed evidence regarding feasibility or effectiveness. Thus, the intervention proposed in the present study represents a significant advancement as it is both a native smartphone app and has undergone scientific validation, which distinguishes it as a robust tool for improving worker well-being.

According to a meta-analysis of work engagement interventions [12], most personal resource-building interventions were delivered in group or face-to-face sessions (eg, [25-27]), which had a significant effect compared to online sessions [22] and the advantage of direct instruction and interaction with others. However, they also have the disadvantage of requiring coordination of time and place and trained staff. One way to overcome these disadvantages is to use smartphone apps. Smartphone apps offer several advantages, such as being easy to start and complete in a short time, being intuitive with minimal explanation, and being ubiquitous without worrying about time and place, offering just‐in‐time interventions [28]. Bartels et al [29] found that mobile technologies are suitable for flexible self-monitoring in everyday life. However, while there are numerous smartphone apps designed to enhance occupational health, safety, and well-being in workplaces, only a limited number of them have been scientifically evaluated [30]. Therefore, it is worthwhile to conduct an evaluation of the effects of a newly developed smartphone-based app.

### Aim

The present study aimed to evaluate the effect of a newly developed smartphone-based positive reflection diary, the Work Engagement Diary (WEDiary), to improve work engagement as a primary outcome among Japanese workers using a randomized controlled design.

## Methods

### Recruitment

Participants were recruited from those registered with a web survey company (Macromill Inc., Tokyo, Japan). Target participants were workers who met the following four inclusion criteria: (1) aged from 20 to 59 years, (2) had two consecutive days off in a week, (3) were not going to quit or change jobs within the next 3 months, and (4) used iOS 10.0 or later or Android 5.1 or later smartphones. First, candidates who were registered with the web survey company received an email invitation from the survey company that included study information and an informed consent form. Respondents read the research explanation, and those who agreed to participate in the study clicked on the “Agree” button on the webpage to indicate their consent. Subsequently, candidates who met the inclusion criteria and agreed to participate in the study were asked to complete a web-based baseline survey. This survey was continued until the number of participants reached 600. The baseline survey began on October 31, 2019, and ended on November 5, 2019. After completing the baseline survey, participants received an email informing them of their allocation group: the intervention group or the wait-list control group. At the end of the study period, participants received redeemable points from the web survey company that could be used for shopping as a reward for participating in the study.

### Sample Size

A sample size of 394 participants per group was calculated, entering α error probability of .05, power (1–β) of 0.80, and small effect size of 0.20 [12,31] using G*Power 3.1 [32]. However, funding constraints led to a sample size of 300 per group.

### Trial Design and Randomization

We conducted a two-group parallel-group, nonblinded, randomized controlled trial (RCT). The study protocol was registered at the University Hospital Medical Information Network Clinical Trials Registry (UMIN000038430). After the baseline survey, participants were randomly allocated to either the intervention group or the wait-list control group at a 1:1 ratio at the individual level by the survey company. The survey company was asked to generate the allocation table and register and assign participants. The researchers were not involved in any of the following activities undertaken by the survey company: random allocation sequence generation and implementation and allocation concealment. The log data of the WEDiary were managed by NT, which were subsequently passed on to MT without being processed. In addition, NT did not have access to the questionnaire data at any of the three time points. Therefore, they were unable to link the log data to the questionnaire data at any of these time points. The study protocol was written according to the Consolidated Standards of Reporting Trials checklist [33].

### Development of Smartphone-Based App

### Overview

**Figure 1.**
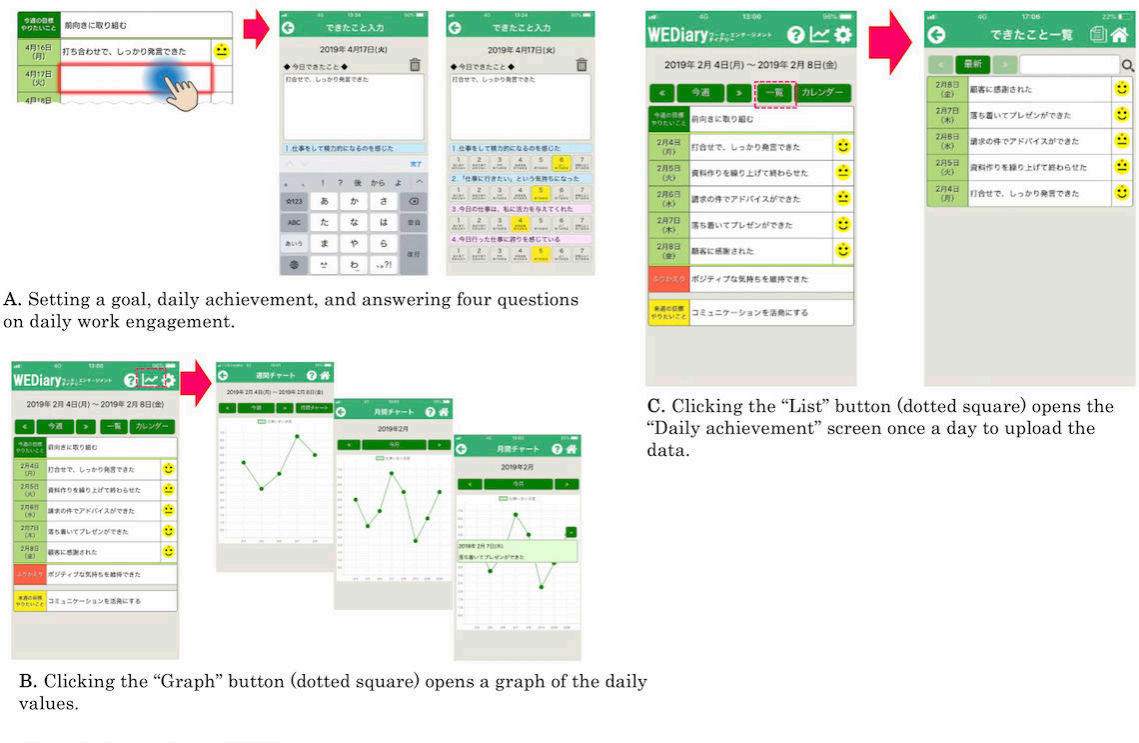
Screenshots of the Work Engagement Diary (WEDiary).

We developed a smartphone-based positive reflection diary app (the WEDiary) for both Android and iOS. Figure 1 shows screenshots of the WEDiary. The intervention period was 2 weeks from November 11 to 24, 2019. We determined the intervention and follow-up periods based on a meta-analysis conducted by Vîrgă et al [23] who found a larger effect size for a 2-week intervention (*d*=0.55, *P*<.05) compared to those of other durations. The meta-analysis also revealed that follow-ups within 1 month postintervention showed a larger effect size (*d*=0.34, *P*<.05) than those conducted at longer intervals. The WEDiary is designed to incorporate a post-work prompt, “Please write about what you were able to do at work today,” aimed at fostering positive reflection on daily work activities. Through consistent engagement with this reflective practice, participants are expected to cultivate and accumulate positive emotions over time. According to the Broaden-and-Build theory, positive emotions expand individuals’ attention and cognitive flexibility, thereby facilitating the generation of diverse ideas and the development of critical personal resources [34]. However, the transient experience of isolated positive emotions alone is insufficient for resource building; instead, sustained accumulation of such emotions is essential [35,36].

On the morning of the first day, participants in the intervention group were required to set a goal for that week by filling in the relevant box in the WEDiary (Figure 1A). At the end of the working day, they filled in “Daily achievement” (serving as a daily reflective diary) and rated the following four questions on daily work engagement, using a 7-point scale ranging from 1 (not at all) to 7 (very well): (1) “I felt energetic at work today (for vigor),” (2) “I felt like going to work (for vigor),” (3) “I was inspired by my work today (for dedication),” and (4) “I am proud of the things I did at work today (for dedication)” (Figure 1). These four questions were adopted to measure daily work engagement based on previous diary studies [10,36,37]. Vîrgă et al.’s [23] meta-analysis of the effects of controlled interventions on work engagement found that the effect size for absorption was small (*d*=0.21, *P*<.01) as opposed to those for vigor (*d*=0.36, *P*<.01) and dedication (*d*=0.35, *P*<.01). Thus, we adopted two items each for “vigor” and “dedication” but none for “absorption.” A line graph of the daily values was available when users clicked the button with the graph icon (Figure 1B). The line graph helped participants enhance positive reflections from their visual perspective. On the last day of the 5 working days, they filled in their weekly reflections and goals for the next week in the relevant box. Finally, they pressed the “List” button on the home screen to open the “Daily achievement” screen once a day (Figure 1C) to check their use of the WEDiary and to upload the data to the server. Empirical evidence supporting this notion includes a study employing the experience sampling method, which demonstrated that positive emotional experiences are associated with enhanced hope and increased work engagement [36]. The WEDiary allows users to record, accumulate, visualize, and reinforce these positive emotional experiences. Complementary findings from a meta-analysis [17] highlight the positive association between post-work positive thoughts and work engagement (ρ=0.493, 95% CI 0.407-0.579) as well as between positive reflection and engagement (ρ=0.482, 95% CI 0.390-0.573). Additionally, positive rumination about work was found to significantly contribute to the accumulation of positive emotions (ρ=0.610, 95% CI 0.51-0.709). The WEDiary’s ability to graphically track work engagement is aimed to further reinforce the accumulation of positive emotional experiences. However, the act of responding to the prompt “What did you do well at work today?” could potentially introduce stress for some users. To tackle this, the WEDiary emphasizes cultivating a habitual focus on positive recollection rather than limiting reflection to specific daily achievements. The tool encourages users to acknowledge progress even if predetermined goals are not fully attained. Additionally, guidance provided within the WEDiary directs users to concentrate on the positive aspects of their experiences, with the option to discontinue its use at any time. Its comprehensive design aims to support the sustainable enhancement of positive emotional states and work engagement, contributing to long-term personal resource development.

#### Intervention Group

A few days before starting the program, participants in the intervention group received an email including the URL/QR code of the WEDiary download website/app store and were required to install the app on their smartphones. Furthermore, on the morning of the first day of the intervention period, they received an email to start the program with user IDs and authentication codes for activating the WEDiary. User registration consisted of three steps. First, participants entered their user IDs, gender, year of birth, and authentication code. Subsequently, they were asked to read and agree with the privacy policy. Finally, they were asked to enter their starting and ending business days for a week. Participants received emails requesting them to download, install, and activate the WEDiary if activation of the WEDiary was not confirmed. Participants in the intervention group received email reminders to use the app every day during the intervention period.

#### Wait-List Control Group

Participants in the wait-list control group were requested to be as usual until the completion of the survey 3 weeks after the intervention. They could use the WEDiary after the 3-week follow-up survey period had concluded, that is, on December 23, 2019, or later.

### Measures

#### Data Collection

The intervention period was from November 11 to 24, 2019. All participants in both groups were asked to complete web-based self-report surveys thrice to measure the effects of the 2-week intervention. The preintervention survey period was from October 31 to November 5, 2019 (T1); the postintervention survey period after the intervention was from November 25 to December 5, 2019 (T2); and the 3-week follow-up survey period was from December 16 to 22, 2019 (T3). Participants were not allowed to proceed to the next question unless they answered the previous question.

#### Primary Outcome

As the primary outcome, work engagement was measured using the Japanese version of the Utrecht Work Engagement Scale (UWES-J), which was reported to be reliable and valid [38]. The UWES-J consists of three subscales of three items each (eg, vigor: “At my work, I feel bursting with energy;” dedication: “My job inspires me;” and absorption: “I get carried away when I am working”). Each item was assessed on a 7-point Likert scale ranging from 0 (never) to 6 (always). The scale score was calculated by adding all the scores and dividing them by the number of items, ranging from 0 to 6. A higher UWES-J score indicated a higher degree of work engagement.

#### Demographic Characteristics

Gender, age, marital status, education, job, employment status, working hours per week during the past month, and years of employment tenure were collected at T1. Only education was collected at T2 because we missed out on collecting it at T1.

### Statistical Analysis

Descriptive statistics for participants’ characteristics at T1 were calculated to identify the differences between the intervention and wait-list control groups. *T*-tests were conducted for continuous variables such as age, working hours per week, and years of employment tenure. *χ*^2^ tests were conducted for categorical variables such as gender, marital status, education, and job. The mean scores and SDs of work engagement at the three time points were calculated.

We conducted intention-to-treat (ITT) and per-protocol analyses (not adjusted) to evaluate the effect of the WEDiary using a mixed model for repeated measures conditional growth model analyses with unstructured covariance and full information maximum likelihood estimation for the data with missing values, respectively. A group (intervention and wait-list control)×time (T1, T2, and T3) interaction was analyzed as an indicator of the intervention effect. Since some participants did not consistently use the WEDiary at the three time points, only those who used it every working day during the intervention period (2 weeks), excluding weekends, and responded to all three surveys were analyzed for per-protocol analysis. In addition, a mixed model for repeated measures conditional growth model analysis was conducted for subgroup analyses, dividing each of the following into two groups: work engagement, age, and working hours (divided into two groups based on their median values); gender (male/female); educational background (university graduate or higher/high school graduate or lower); white-collar/non-white-collar; and employment status (regular employees/nonregular employees).

The effect sizes (95% CIs) were calculated using Cohen *d* only among the participants for per-protocol analysis, although the effect sizes may be biased due to dropouts. Values of 0.2, 0.5, and 0.8 are generally interpreted as small, medium, and large effects, respectively [39].

To evaluate the process evaluation, the number and percentage of those who installed the WEDiary and active users of the WEDiary were calculated. Active users were defined as those who clicked the “List” button to upload the diary data, such as “Daily achievement,” and rated the four questions on daily work engagement. However, those who clicked the “List” button without uploading the diary data or did not rate the four questions were excluded.

To evaluate the trajectories of the four questions in the WEDiary for 10 days (excluding weekends), a mixed model analysis was conducted using the total score of the four questions as the dependent variable and time as the independent variable.

Two-tailed test with a *P* value set to .05 was used in all statistical analyses. All statistical analyses were performed using the Statistical Package for Social Sciences for Mac version 27 (IBM Corp., Armonk, NY, USA).

### Ethical Considerations

This study was reviewed and approved by the Research Ethics Review Board of the Faculty of Policy Management and the Faculty of Environment and Information Studies at Keio University (approval no.: 265), in accordance with the Declaration of Helsinki and relevant ethical guidelines. All participants were provided with detailed information about the study on a designated web page, including its purpose, procedures, potential benefits, and their rights. Informed consent was obtained electronically from those who agreed to participate. Participation was entirely voluntary, and participants were free to withdraw from the study at any time without penalty. To ensure privacy and confidentiality, all questionnaire data were collected by a survey company, anonymized (decoded), and subsequently provided to the researchers. Personally identifiable information was retained solely by the survey company and was not accessible to the research team. Participants had the opportunity to benefit directly, as the intervention had the potential to improve work engagement. In addition, participants received a reward of points equivalent to 1000 yen (approximately US $9 at the time of the study), which could be used for shopping upon completion of the study.

## Results

### Participants, Randomization, and Attrition

**Figure 2.**
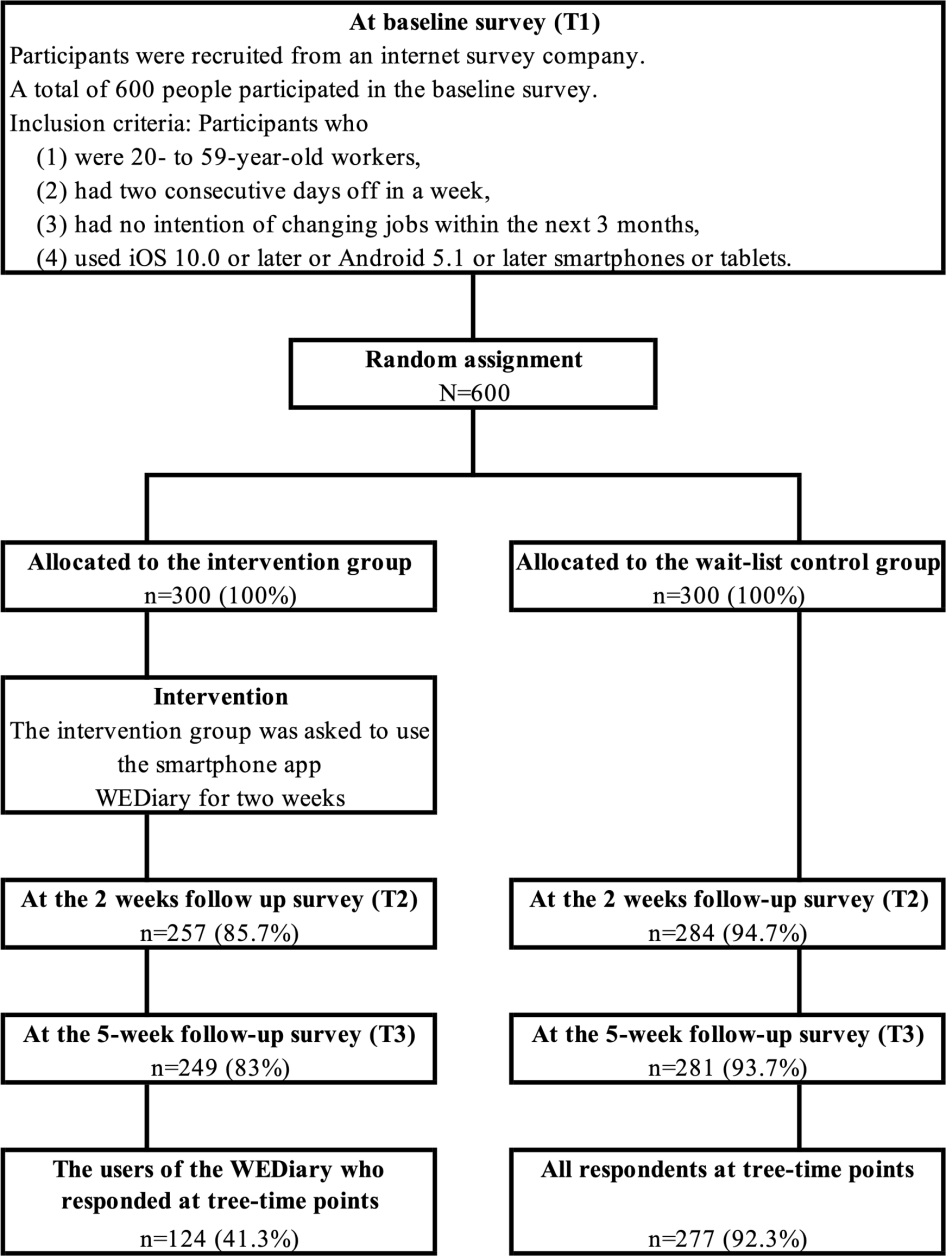
Participant flowchart.

Figure 2 presents the flowchart and randomization of the participants. After the baseline survey, 600 participants were randomly allocated to either the intervention group or the wait-list control group (300 participants per group). After the 2-week intervention period, participants answered the questionnaires again at T2 and at T3. The number of respondents in the intervention group was 257 (85.7%) at T2 and 249 (83%) at T3, while that in the wait-list control group was 284 (94.7%) at T2 and 281 (93.7%) at T3.

### Demographic Characteristics

Table 1 presents participants’ demographic characteristics at T1. There were no significant differences in characteristics between the intervention and wait-list control groups, except for education. Most participants were male (the intervention group: 68.3%; the control group: 67%) and married (intervention group: 62%; control group: 65.7%). Education was significantly different between the intervention and wait-list control groups (*P*<.001). More participants in the intervention group, as opposed to the wait-list control group, had completed high school or less (19.7% for the intervention group and 15.7% for the control group) and graduate school (8.3% for the intervention group and 7.7% for the control group).

**Table 1.** Baseline characteristics of participants in the intervention and wait-list control groups.

	Intervention group (n=300)	Wait-list control group (n=300)	*P* value^a^
Gender, n (%)			.73
Male	205 (68.3)	201 (67)	
Female	95 (31.7)	99 (33)	
Age, mean (SD)	40.76 (9.5)	41.69 (10.3)	.25
Marital status, n (%)			.35
Married	186 (62)	197 (65.7)	
Unmarried	114 (38)	103 (34.3)	
Education^b^, n (%)			<.001
Under high school	59 (19.7)	47 (15.7)	
College	41 (13.7)	48 (16)	
University	130 (43.3)	166 (55.3)	
Graduate school	25 (8.3)	23 (7.7)	
Other/don’t want to answer	2 (0.7)	0 (0)	
N/A^c^	43 (14.3)	16 (5.3)	
Job, n (%)			.56
Professional/engineer	68 (22.7)	73 (24.3)	
Manager	34 (11.3)	47 (15.7)	
Clerical	91 (30.3)	86 (28.7)	
Sales	37 (12.3)	30 (10)	
Service	11 (3.7)	11 (3.7)	
Production/skills	28 (9.3)	31 (10.3)	
Security officer	2 (0.7)	3 (1)	
Forestry/fishery	1 (0.3)	0 (0)	
Transportation/communications	7 (2.3)	2 (0.7)	
Others	21 (7.0)	17 (5.7)	
Employment status, n (%)			.37
Executive officer	6 (2)	3 (1)	
Self-employed	4 (1.3)	2 (0.7)	
Self-employed (help)	1 (0.3)	0 (0)	
Regular employee	261 (87)	259 (86.3)	
Part-time employee	5 (1.7)	2 (0.7)	
Temporary employee	9 (3)	14 (4.7)	
Contract employee	11 (3.7)	19 (6.3)	
Others	3 (1)	1 (0.3)	
Working hours per week, mean (SD)	41.5 (15.5)	41.6 (17.1)	.92
Employment years, mean (SD)	11.5 (9.5)	12.5 (10.7)	.23

a *t* test or *χ* test.

b Measured at T2.

c N/A: not available.

### Outcome Mean Scores and SDs

Table 2 shows the means and SDs of the outcome variables at T1, T2, and T3 in the intervention and wait-list control groups. The means (SDs) for the intervention group were 2.6 (1.1) at T1, 2.7 (1.1) at T2, and 2.7 (1.2) at T3. The means (SDs) for the wait-list control group were 2.6 (1.1) at T1, 2.6 (1.1) at T2, and 2.5 (1.2) at T3. The reliability estimated by Cronbach α was .93 at T1, .95 at T2, and .95 at T3 for this study.

**Table 2.** Means (SDs) of outcome variables at T1, T2, and T3 in the intervention and wait-list control groups for the whole sample.

	Intervention group		Wait-list control group	
	Participants, n	Mean (SD)	Participants, n	Mean (SD)
T1	300	2.6 (1.1)	300	2.6 (1.1)
T2	257	2.7 (1.1)	284	2.6 (1.1)
T3	249	2.7 (1.2)	281	2.5 (1.2)

### Effects of the WEDiary

#### Intention-to-Treat Analyses

Table 3 shows the estimated effect of the WEDiary on work engagement based on the mixed model analyses for the ITT analyses. There was a significant effect on work engagement between the two groups (*t*=2.03, *P*=.04). For the subscales, significant effects on vigor (*t*=2.30, *P*=.02) and dedication (*t*=2.78, *P*=.01) were found, although no significant difference on absorption (*t*=0.35, *P*=.73) was found between the two groups.

**Table 3.** Result of mixed model for repeated measures conditional growth model analyses for the whole sample throughout the study period.

	Effect	SE	*df*	*t*	*P* value	95% CI (lower to upper)
Work engagement	0.07	0.03	545	2.03	.04	0.00 to 0.13
Vigor	0.08	0.04	549	2.30	.02	0.01 to 0.16
Dedication	0.11	0.04	546	2.78	.01	0.03 to 0.18
Absorption	0.01	0.04	546	0.35	.73	–0.06 to 0.09

#### Per-Protocol Analyses

In per-protocol analyses, the number of participants was 124 for the intervention group and 277 for the wait-list control group. A significant effect on work engagement (*t*=2.61, *P*=.01) was found between the two groups. For subscales, significant effects on vigor (*t*=2.32, *P*=.02) and dedication (*t*=3.26, *P*=.001), but not on absorption (*t*=1.22, *P*=.22), were found between the two groups (Table 4).

**Table 4.** Effect of the Work Engagement Diary (WEDiary) on work engagement between the intervention group using the WEDiary for every working day (n=124) and the wait-list control group (n=277).

	Effect	SE	*df*	*t*	*P* value	95% CI (lower to upper)
Work engagement	0.11	0.04	399	2.61	.01	0.03 to 0.19
Vigor	0.11	0.05	399	2.32	.02	0.02 to 0.20
Dedication	0.15	0.05	399	3.26	.001	0.06 to 0.24
Absorption	0.06	0.05	399	1.22	.22	–0.04 to 0.16

### Subgroup Analyses

A mixed model analysis was conducted on work engagement as the dependent variable, dividing each of the following into two groups: work engagement, gender, age, working hours, educational background, white-collar/non-white-collar, and employment status. The results showed that work engagement significantly improved among females, university graduates or higher, white-collar workers, and regular employees (*t*=2.85, *P*=.005; *t*=2.37, *P*=.02; *t*=2.37, *P*=.02; *t*=2.37, *P*=.02, respectively).

### Effect Size

The effect size was small at each time point (T1 vs T2 and T1 vs T3; Table 5). The number of questionnaire respondents at both T1 and T2 was 257 for the intervention group and 284 for the wait-list control group, respectively, whereas that at both T1 and T3 was 249 for the intervention group and 281 for the wait-list control group, respectively.

**Table 5.** The effect sizes of the Work Engagement Diary (WEDiary).

	T1 versus T2	T1 versus T3
	Cohen *d* (95% CI [lower to upper])	Cohen *d* (95% CI [lower to upper])
Work engagement	0.11 (−0.06 to 0.28)	0.12 (−0.06 to 0.28)
Vigor	0.06 (−0.11 to 0.23)	0.14 (−0.03 to 0.31)
Dedication	0.16 (−0.02 to 0.32)	0.17 (−0.002 to 0.34)
Absorption	0.06 (−0.11 to 0.23)	0.01 (−0.16 to 0.18)

### Process Evaluation

**Figure 3.**
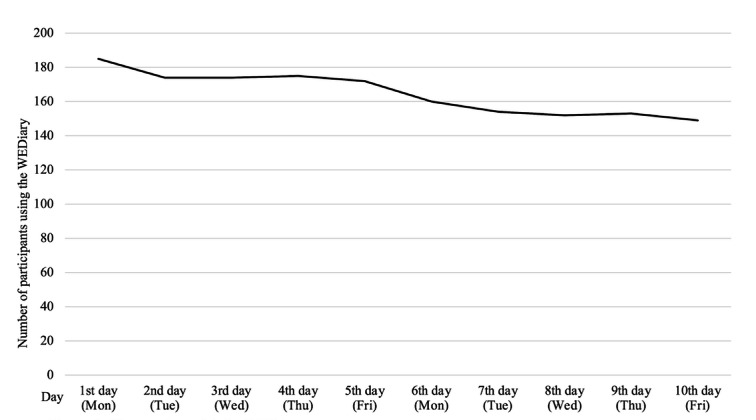
The number of daily WEDiary users.

Of the 241 (80.3%) participants in the intervention group (n=300) who installed the WEDiary on their smartphones, 191 (63.7%) used it at least once. The number of daily users gradually decreased during the intervention period. The first day of the intervention period had 185 users (61.7%), which was the highest number. In contrast, the number of users on the last day of the intervention period was 149 (49.7%), which was the lowest (Figure 3).

### Trajectories of the Four WEDiary Questions

The total scores of the four questions included in the WEDiary were analyzed using a mixed model (linear regression model), with the total score as the dependent variable and the time point as the independent variable. The intercept (mean value) at the 10 time points was 3.58, and the slope was 0.035 (*P*<.01), showing a significant increase over time. Each of the four items also had a significant positive slope; however, the slope parameters were small.

## Discussion

### Effect on Work Engagement

The present study showed that our newly developed smartphone-based positive diary app for enhancing work engagement (WEDiary) among Japanese workers had a significantly favorable effect on work engagement in the intervention group compared to the wait-list control group during the study period. Thus, the WEDiary was effective in improving work engagement among participants. Subgroup analysis showed that work engagement significantly improved among females, university graduates or higher, white-collar workers, and regular employees. This suggests that providing the WEDiary to specific target groups is effective. Females tend to ruminate more than males in response to stress [40]. WEDiary may have guided female participants toward positive rumination (ie, positive reflection). This result can also be explained by the Broaden-and-Build theory, which posits that positive emotions expand individuals’ attention and cognitive flexibility, thereby facilitating the generation of diverse ideas and the development of critical personal resources [34]. Participants with a university degree or higher are likely to be self-regulated learners and tend to be highly capable of utilizing self-efficacy [41]. As a result of using WEDiary and enhancing self-efficacy as a personal resource, work engagement may have increased. The JD-R model is a valid theoretical framework regardless of job type; however, the importance of specific demands and resources may vary depending on the job type [42]. In this study, in terms of enhancing personal resources, white-collar workers may have benefited more from using WEDiary compared to non-white-collar workers. Future research should examine job types rather than the two categories of white-collar and non-white-collar workers to potentially refine the WEDiary. Furthermore, in the JD-R model, nonregular employees may represent a vulnerable group with fewer job resources [43], while regular employees, who typically possess relatively higher job resources, may experience greater enhancement of personal resources through using WEDiary. Thus, the WEDiary, which focuses on one’s strengths and positive experiences at work, is likely to be particularly effective for females with a high tendency to ruminate, highly educated individuals with self-control, white-collar workers, and regular employees possessing high job resources.

### Effect on Vigor and Dedication

The WEDiary might have facilitated participants to gain a greater sense of achievement, such as through “Daily achievement” (serving as a daily reflective diary) or the visualized line graph, leading to enhanced work engagement. Participants set a weekly goal at work, filled in one daily achievement at work, and answered four questions about work engagement. A text box was provided to indicate daily achievement in one short sentence. Answering the four questions about work engagement and the text input of what was accomplished provided an opportunity to reflect on the day’s work. Through this process, participants may gain a greater sense of achievement at work, leading to improved work engagement [17]. In other words, this process may enhance the levels of energy and mental resilience in one’s work (vigor) and strong involvement in one’s work combined with a sense of significance, enthusiasm, and challenge (dedication).

On the other hand, absorption (ie, having higher concentration, being happily engrossed in one’s work, and being in a state of mind in which time seems to pass quickly and in which it is difficult to leave work) may have had delayed effects. The fact that the WEDiary was used at the end of the working day may be why the effect on absorption was not immediately evident. Future research with a longer study period than that of the current study may have more success in observing the effect on absorption. Continuous use of the WEDiary may improve work engagement and, in the long run, absorption. Another possibility is that the WEDiary may have a low effect on absorption. Prior meta-analyses of studies on work engagement improvement also found absorption to be lower than vigor and dedication [23,44]. It may therefore be necessary to consider an improved version of the WEDiary that takes absorption improvement into account.

### Comparison With Prior Work

In comparison to prior interventions focusing on building personal resources, the strength of our study lies in targeting diverse workers (hotel employees [45], caregivers [45]), with work engagement (exhaustion [45,46]) as its outcome. Hence, the generalizability of the WEDiary may be higher compared to prior interventions focusing on building personal resources. Vîrgă et al’s [23] meta-analysis on the effectiveness of interventions intended to improve work engagement found a modest but significant overall improvement in work engagement (*d*=0.21, *P*<.01), with notable gains in vigor (*d*=0.36, *P*<.05) and dedication (*d*=0.35, *P*<.05), though absorption showed a smaller effect (*d*=0.16, *P*<.05). In addition, it was found that intervention effects peaked within the first month (*d*=0.34, *P*<.05) but declined over time, becoming negligible by 6‐12 months. Interventions of up to 2 weeks (*d*=0.55, *P*<.05) or those between 12 and 16 weeks (*d*=0.46, *P*<.05) were deemed most effective, while very short or extended interventions were less impactful. The WEDiary, a smartphone app based on positive psychology, aims to improve work engagement through a 2-week intervention and a 3-week follow-up, suggesting potential effectiveness based on these findings.

### Time of Appearance of the Effect

Effect sizes were relatively larger for T1 versus T3 than for T1 versus T2 (except for absorption). This could be a delayed effect, which may be a sleeper effect [47]. It occurs when an intervention does not have an immediate effect and requires incubation time to produce its effect [47]. It may take time for work engagement to improve through the use of the WEDiary. Regarding previous studies on improving work engagement by focusing on personal resources, those with an intervention period of 30 days [48] and 8 weeks [49] significantly improved work engagement at postintervention, while those with an intervention period of 2 weeks [25] from baseline to follow-up survey and half a day [50] from baseline to 1-month follow-up showed no statistically significant effects. On the contrary, a meta-analysis of intervention studies aimed at improving work engagement [23] reported that the effect size for a 2-week intervention period was relatively high at *d*=0.55 (*P*<.05) compared to other intervention periods and that the effect size for a follow-up period of less than 1 month was relatively higher (*d*=0.034, *P*<.05) than those for other periods (1‐3 months: *d*=0.28; 3‐6 months: *d*=0.07; 6‐12 months: *d*=0.03; all *P*<.05). The strength of the present study is that the WEDiary with a 2-week intervention period showed a sustained significant effect throughout the study period of 4 weeks. The intervention and follow-up periods of the WEDiary may have been suitable for evaluating the effect. However, while the intervention aimed at encouraging work engagement improved two of the three subdimensions of work engagement (ie, vigor and dedication), improvements in the third subdimension (ie, absorption) remained limited. This result is in line with recent research that has reported a similar trend. There are several possible reasons for the limited improvement observed in absorption, including work environment characteristics [51] and individual intrinsic motivation [52]. Therefore, it is suggested that the approach to improving immersion should be based not only on psychological intervention but also on a thorough review of work design and the interests and concerns of individual employees.

### Small Effect Size

Unfortunately, the effect size was small in this study. However, despite this, effect sizes were relatively larger for T1 versus T3 (Cohen *d*=0.12) than for T1 versus T2 (Cohen *d*=0.11). This may be because it takes time to accumulate positive emotions. A prior meta-analysis of interventions aimed at improving work engagement [23] reported effect sizes of *d*=0.14 (95% CI 0.05-0.22) and *d*=0.29 (95% CI 0.16-0.41) for online and face-to-face interventions, respectively. Considering that the WEDiary is a self-learning program with no feedback function and that the intervention period was 2 weeks long, an effect size of 0.11 immediately after the intervention and 0.12 at follow-up is in line with previous studies and suggests the possibility of participants being in the middle of accumulating positive emotions. This could be because the WEDiary usage rate gradually decreased during the intervention period (2 weeks); users who used the app infrequently or not at all may have weakened the effect of the intervention. Allocated interventions such as the RCT have a smaller effect than volunteered interventions [11]. This speculation could be supported by the fact that the intervention effect improved in the per-protocol analyses compared to ITT analyses. In the future, we need to investigate how to enhance the installation and use of an app, such as using push notifications or messages. However, the significance level was set at *α*=.05 in this study, and there is a possibility that the statistical power (1–β) may be low, necessitating caution when interpreting the results. A low power increases the risk of Type II errors, potentially leading to false-negative conclusions. Additionally, it is essential to assess whether the effect size used in the sample size calculation (Cohen *d*=0.20) was appropriate, particularly when comparing conditions such as face-to-face versus online formats or the presence versus absence of support. An underestimated or overestimated effect size could impact the validity of the findings and the robustness of the statistical conclusions. Increasing sample sizes has recently become a critical concern in online psychological interventions aimed at behavior change. Previous studies have identified several effective strategies for expanding participant pools. Utilizing digital platforms, such as chatbots, has significantly enhanced sample sizes by improving accessibility and user engagement [53]. Additionally, large-scale online recruitment methods, including the use of social media and targeted advertising, have proven effective in reaching diverse populations and increasing the number of participants [54].

### User Attrition

Process evaluation clearly showed user attrition. Positive psychology interventions such as the one used in our study, in particular, often have low participation rates and high attrition [11]. In a positive psychology study involving workers similar to those in the present study, attrition may have been due to participants perceiving the required time commitment as burdensome and to the absence of face-to-face interactions, as WEDiary was self-learning [55]. The following solutions to user attrition have been examined: self‐selection [56], participant autonomy [57], and belief [58]. To apply our results to different work settings, the WEDiary with the addition of the above strategies may have the potential to increase the promotion of use. This study did not include a qualitative survey on user experience such as satisfaction. Further detailed qualitative surveys on user experience are needed in the future. This may contribute to future app updates.

### Questionnaire Response Rate

The questionnaire response rate was higher in the wait-list control group than in the intervention group (85.7% vs 94.7% at T2, 83% vs 93.7% at T3). This may be because the expectation of using the WEDiary in the wait-list control group may have encouraged the participants to respond to the questionnaires. In this study, data from the wait-list control group were not collected. However, including a plan to collect and analyze data from the wait-list control group after using the app in such intervention studies may help clarify the mechanisms of improvement in outcomes such as work engagement in more detail.

### Limitations

The present study has several limitations. First, the participants were all registered members of the online research company, which requires caution regarding the generalizability of our findings. Second, internet users’ socioeconomic and educational status is usually high on average, compared to that of the general population [59,60]. Indeed, participants in the present study had a higher educational status than those completing nationwide paper-and-pencil surveys in Japan [61]. Third, the small sample size compared to the calculated sample size might have lowered statistical power compared to expectations. The sample size of the study was 300 participants per group compared to the estimated number of 394 participants per group needed to detect an effect size of 0.20 for work engagement. Thus, this study had a lower statistical power. Fourth, the low adherence rate to the study protocol may have weakened the effect of this study. Of the 300 participants, 241 (80.3%) installed the app but only 191 (63.7%) used the WEDiary. Previous studies on smartphone-based interventions did not indicate the installation rate because it was required that the smartphone app be installed in advance [62-64]. To evaluate the effects of the intervention, it may be necessary to require participants to download and install the WEDiary prior to the presurvey. Alternatively, we may need to develop strategies to encourage participants to download and install the WEDiary actively. Further research is required to increase the installation rate. Fifth, participants with social desirability or the dropout of nonresponders might cause a biased estimate. Sixth, this study employed a nonblinded RCT design, which may introduce potential bias in both the intervention and wait-list control groups. Seventh, it is possible that work engagement measured using the UWES-J at the three time points correlates with the four questions included in the WEDiary (modified UWES-J). In other words, responding to the UWES-J at T2 and T3 may lead to improving positive emotions in the participants. However, UWES-J items use the present tense to measure the present state, with no items reflecting on the state of only the day. The UWES-J may be unlikely to improve work engagement. Furthermore, of the four WEDiary questions, three include the word “today;” hence, each of these three questions asks for an evaluation of today. Eighth, this study analyzed the positive motivational process of the two processes of the JD-R model. The analysis did not control for factors such as the job demands and job resources of the JD-R model. No control for factors could limit the accurate determination of the effectiveness of the intervention and the potential buffering or mediating effects of these variables; hence, further research is needed to clarify the mechanisms.

Finally, there are limitations in the generalizability of the results because the present study was conducted for Japanese workers. It is unclear whether similar results can be obtained for non-Japanese workers. Further research is needed for a variety of workers.

### Conclusions

This RCT demonstrated that our newly developed smartphone-based positive reflection diary (WEDiary) was effective throughout the three time points in improving work engagement among Japanese workers. Effect sizes were relatively larger for T1 versus T3 than for T1 versus T2. Future research needs to clarify longer-term intervention effects and detailed mechanisms of the intervention effects.

## Supplementary material

10.2196/55664 Checklist 1 CONSORT-EHEALTH (V 1.6.1) checklist.

## Abbreviations

ITT: intention-to-treat
JD-R: job demands–resources
RCT: randomized controlled trial
UWES-J: Utrecht Work Engagement Scale-Japanese version
WEDiary: Work Engagement Diary
